# Interfacial Micromechanics in Fibrous Composites: Design, Evaluation, and Models

**DOI:** 10.1155/2014/282436

**Published:** 2014-04-07

**Authors:** Zhenkun Lei, Xuan Li, Fuyong Qin, Wei Qiu

**Affiliations:** ^1^State Key Laboratory of Structural Analysis for Industrial Equipment, Dalian University of Technology, Dalian 116024, China; ^2^Department of Mechanics, Tianjin University, Tianjin 300072, China

## Abstract

Recent advances of interfacial micromechanics in fiber reinforced composites using micro-Raman spectroscopy are given. The faced mechanical problems for interface design in fibrous composites are elaborated from three optimization ways: material, interface, and computation. Some reasons are depicted that the interfacial evaluation methods are difficult to guarantee the integrity, repeatability, and consistency. Micro-Raman study on the fiber interface failure behavior and the main interface mechanical problems in fibrous composites are summarized, including interfacial stress transfer, strength criterion of interface debonding and failure, fiber bridging, frictional slip, slip transition, and friction reloading. The theoretical models of above interface mechanical problems are given.

## 1. Introduction

Polymer-matrix fibrous composites have been widely used in the aerospace and industrial locomotives fields. There exist many interfacial phenomena and all kinds of defects including inclusions, pores, and layer shrink zones in fibrous composites during design and manufacturing processes. Fiber fracture and interface debonding will appear inside the material in service and result in early fatigue, aging, damage, and failure, so it is a hidden danger to risk a major engineering accident. With development of composite material science and aerospace industry applications, the light-weight and high-tough carbon fibers have been widely applied to fibrous composites. Therefore, many researchers coming from physics, chemistry, materials, mechanics, and engineering are attracted by many basic mechanical problems in fibrous composites, such as mechanical properties characterization of high-performance fiber, fiber/matrix interface debonding, fiber bridging, fiber fracture, and matrix cracking [1].

Besides the effects of fiber surface treatments on fibrous composites have been studied; the basic problems of microscopic interfacial stress transfer and failure were focused in decades [2, 3]. Materials' microstructure configuration determines its response to external action. As a connection between reinforcing fiber and matrix, the interface is an important microstructure of fibrous composites including fiber, fiber transition region, fiber surface coating, matrix transition region, and matrix. The interface is a bridge connecting both reinforced fiber and matrix and a deliverer of mechanics information. Although the interface is much smaller than the size of composite bulk, there are many mechanical problems on the interface, such as load transfer, shear strength, interface debonding, damage, and stress singularity [4, 5]. Interfacial bonding quality directly affects the entire composites on interlaminar shear, fracture, impact, heat aging, wave propagation, and other mechanical properties. Therefore, it is necessary to study on fiber interface mechanics from a microscopic view by examining and analyzing the linkages among microstructure, interfacial mechanics properties, and macrofracture properties. The establishment and improvement of interfacial stress transfer and failure models will help to understand the composite stress transfer, debonding, and failure mechanisms from microscale experiments.

With unique advantages of nondestructive, noncontact, and high spatial resolution (1 *μ*m), micro-Raman spectroscopy (MRS) is most likely applied to the integrity characterization of interfacial micromechanical properties in fibrous composites [6], porous silicon [7–9], and carbon nanotubes [10, 11]. In the process of interface debonding of fibrous composites, the evolution of interface mechanical parameters including the frictional shear stress, interfacial shear stress, and debonding length happened in real time, while the pulling force and displacement are also changed accordingly. At present, the main mechanical problems on fiber/matrix interface include the stress transfer, interfacial strength criterion, fiber bridging, and other aspects in fibrous composites.

Based on the outline of interface mechanics design, interface evaluation method, and fine characterization techniques of fibrous composites, the research progress on the interface mechanics by MRS is introduced in the paper, including the interfacial stress transfer, interfacial debonding and strength failure criterion, fiber bridging, interface friction, and slip transition. At last, some theoretical models on those interface mechanics problems are summed up.

## 2. Interface Mechanics Design

The mechanical properties of fibrous composites are closely related to the interface control process, material compound, material properties, and interfacial failure modes, so these are very important for the interface mechanics design and optimization of fibrous composites. However, there is no effective criterion yet to optimize the performance of interface mechanics. Based on the existing interface mechanics models and numerical analysis works, it is possible to get the interfacial mechanical parameters and material parameters having no interface failure and to give the reference of the process controls and material options for the optimization of interface mechanical properties [12, 13].

**Figure 1:**
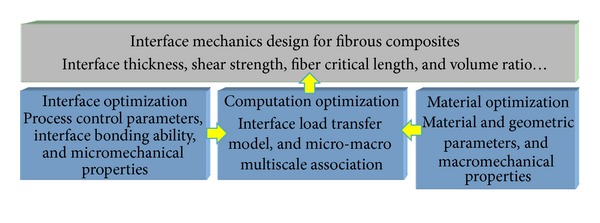
Interface design routes for fiber composites.

The design of interface mechanics in fibrous composites should consider the process technology, materials and environment, and other complex factors. It mainly consists of three optimization approaches: material optimization, interface optimization, and computation optimization, as shown in Figure 1. The former two approaches control the macro- and micromechanical properties of fibrous composite, respectively. Meanwhile, they are helpful to study the interfacial load transfer and failure models for different scales and to provide the theoretical and experimental basis for the interface mechanics design of fibrous composites. The third approach is utilizing the multiscale computation to associate the macro- and micromechanical models and to predict the ultimate bearing performance of the designed fibrous composites through optimizing material constituents and fiber laying configurations.

### 2.1. Material Optimization

The selection and optimum combination of materials is the most commonly used method for the interface design of fibrous composites. Through choosing fiber and matrix resin having specific properties, the composite laminates are formed by curing according to a certain volume ratio and fiber laying manner. Thus, the loading capacity of fibrous composites can be improved by means of the excellent mechanical properties of fibers. Typically, the macroscopic mechanical tests are used to characterize the interfacial properties of fibrous composites. There are a lot of works to get the interfacial shear strength and other interface parameters, such as the fiber critical length obtained by single fiber fragmentation test and the relationship between fiber pullout force and displacement by single fiber pullout test [17, 18].

The interface mechanical parameters obtained by the macroscopic mechanical tests are the average results for characterizing the macroscopic performance of interface bonding capability. However, it is difficult to get the fine stress distribution along the fiber/matrix interface and to observe the interfacial debonding and failure processes. Therefore, the development of microscale measurement methods is necessary [19]. In addition, there are still more researches on the mechanical testing of fiber and resin matrix itself, including tensile or compressive stress-strain behaviors of single fiber and fiber bundle, the impact of fiber surface treatment on the interface shear strength, the resin curing behavior, and wetting behavior between fiber and resin. The impact of these factors on the fiber/matrix interface physicochemical properties and geometric characteristics still needs further study.

### 2.2. Interface Optimization

Nowadays, it has been recognized that the way of traditional compound optimization is insufficient to improve the whole mechanical properties of fibrous composites and then the researchers turned to the interface bonding ability to improve the mechanical properties of fibrous composites. Due to different mechanical properties, compound process, and geometric conditions on the fiber/resin interface, there are thermal, mechanical, chemical, and physical coupling effects existing on the interface. Resultantly, different interfacial structures and characteristics appear and affect the fiber/resin interface bonding capacity, and then the fibrous composites exhibit different macrophysicochemical and mechanical properties [20].

**Figure 2:**
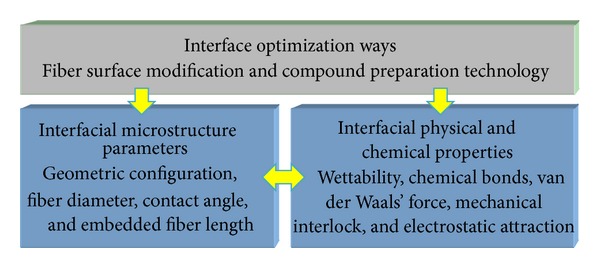
Interface optimization ways.

As shown in Figure 2, the interface control means for fibrous composites are mostly through the fiber surface modification and compound process to obtain specific fiber/matrix interface microstructure, such as the geometric configurations, contact angle, and embedded fiber length. The specific interface microstructure will exhibit different physical and chemical properties, such as wettability, chemical bond, and van der Waals force; thus the interface bond strength is changed to improve the performance of interfacial load transfer. At present, the fiber surface modification can be used to get appropriately bonded interface, but the physicochemical mechanisms that are how to affect the interfacial micromechanical properties as well as how to control the interfacial stress transfer have been concerned. Although a variety of interface theories in fibrous composites have been proposed, such as wettability theory, chemical bond theory, and friction theory, there is no perfect theory to explain all phenomena of interface [2].

### 2.3. Computation Optimization

If the interface strength of fibrous composites is too low, the fiber is easy to debond, pullout, break, and fail. On the contrary, if the interface strength is very high, the stress between the fiber and matrix cannot be relaxed and the brittle fracture would occur at the interface. Therefore, the interface design can be optimized by considering the best comprehensive mechanical properties. The interfacial mechanical properties and geometrical parameters are regarded as design variables, and then certain optimization method such as genetic algorithm combines with the finite element analysis to find the best design variables. This is the fast optimization path of interface mechanical performance in fibrous composites.

However, the design variables of composite interface microstructure are not continuous so that the derivative-type optimization method will fail in the case. It is also noted that the uncertainty of initial value limits the capacity of optimization method converging to the global optimum. In addition, the existing mechanical models are imperfect to describe the micromechanical behavior of the composite interface. The mechanical properties of interface layer, residual stress, and stress singularity are the difficulties to constrain the numerical computation [12, 13]. At present, a lot of works are still to seek the appropriate computing optimization methods to solve such problems. It is inevitable and reasonable way for the computation optimization to perfectly combine with the interface evaluation tests, fine interface characterization techniques, and interface mechanical models.

## 3. Interface Evaluation and Characterization

### 3.1. Interface Evaluation Tests

**Figure 3:**
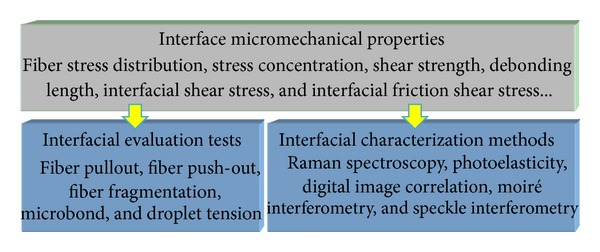
Evaluation tests and characterization methods for micromechanical properties of interface.

The macroscopic damage and failure criteria for fibrous composites do not consider the micromechanical properties of interface, such as fiber stress distribution, stress concentration, shear strength, and frictional shear stress on the debonding interface. In addition, there is still lack of a common understanding about the influence of interfacial microstructural parameters and physicochemical properties on the interface micromechanical properties. Currently, the research on microscale experimental mechanics characterization of the interface failure is not only the most difficult and crucial problem but also the important content of interface mechanical evaluation in fibrous composites, as shown in Figure 3.

The interfacial shear strength is a commonly used parameter to evaluate interfacial bonding quality, fiber/matrix stress transfer efficiency, and the effect of fiber surface modification. The important parameter can be obtained by single fiber micromechanical testing experiments. One kind of these experiments is realized by applying the external load to single fiber, such as fiber pullout test [15], microbond test [21], microdroplet tension test [14, 22], and fiber push-out test [23]. The other is finished by applying the external load to the resin matrix, such as fiber fragmentation test [12, 13] and Broutman test [24].

During the implementation and application of these interface evaluation tests for the characterization of micromechanical properties, it is difficult to ensure the integrity, repeatability, and consistency of the interface evaluation. The experimental results of fiber pullout test, fiber fragmentation test, and fiber push-out test vary widely at the same external conditions. Even using the same test method, the experimental results among different laboratories still have differences [25]. Further studies suggest that this difference comes from the stress singularity at the fiber end [26], so the reevaluation of these test methods and the development of new, more appropriate test methods are concerned [27].

However, the deeper reason is that the differences of many conditions (i.e., interface characteristics) in these interface evaluation tests are neglected, such as interface structure, geometric shape and dimension, and boundary and surface treatment. The testing specimens employed in the different interface evaluation methods have different geometric parameters, such as the droplet contact angle and the embedded fiber length in the microdroplets tension test. Even with the same specimen preparation procedure, it is difficult to ensure that all samples have a uniform geometry and dimension size, which affects the repeatability and consistency of the interface micromechanical parameters characterized by the interface evaluation test. The latest research of microdroplet tension test shows that the microdroplet conformations with different contact angles affect the interfacial shear stress distribution and stress transfer efficiency [28]. By optimizing the design of interface geometry to reduce or even eliminate the stress singularity, the mechanical behavior of fibrous composites can be upgraded [29]. Therefore, the further research on the interface geometry and physicochemical properties affecting the interfacial stress transfer behavior will benefit to optimize the interfacial stress distribution and reduce the stress concentrations.

### 3.2. Fine Characterization Techniques

Commonly, the interfacial shear strength obtained by the interface evaluation tests is used as an important characteristic parameter in the interface failure models and is an average value for characterizing the interface bonding properties. It cannot completely describe the details of the interfacial stress transfer and interfacial debonding failure processes. Therefore, more sophisticated real-time experimental data are required to quantitatively and completely characterize the micromechanical behaviors of the fiber/matrix interface [30]. The direct requirement is the use of “partial details” (i.e., stress distribution) of the interface parameters instead of the average. In addition, the respective contributions of the bonding shear stress and frictional shear stress to the interfacial shear failure mechanisms are also concerned in the fiber/matrix debonding procedure. However, most studies are lacking in the integrity of mechanical description for the procedures of the interfacial stress transfer and interfacial debonding failure. A very important reason is the lack of suitable microscale stress-strain measurement techniques and full-field observation means.

The testing methods having the ability to carry out the microscale fine characterization, including MRS and digital photoelasticity, digital image correlation, and speckle interferometry. These methods are most likely the first application to completely characterize the micromechanical properties of fiber reinforced composites. MRS measurements have unique advantages at the microscales: nondestructive, noncontact, high spatial resolution (1 *μ*m), and the depth focus [6].

**Figure 4:**
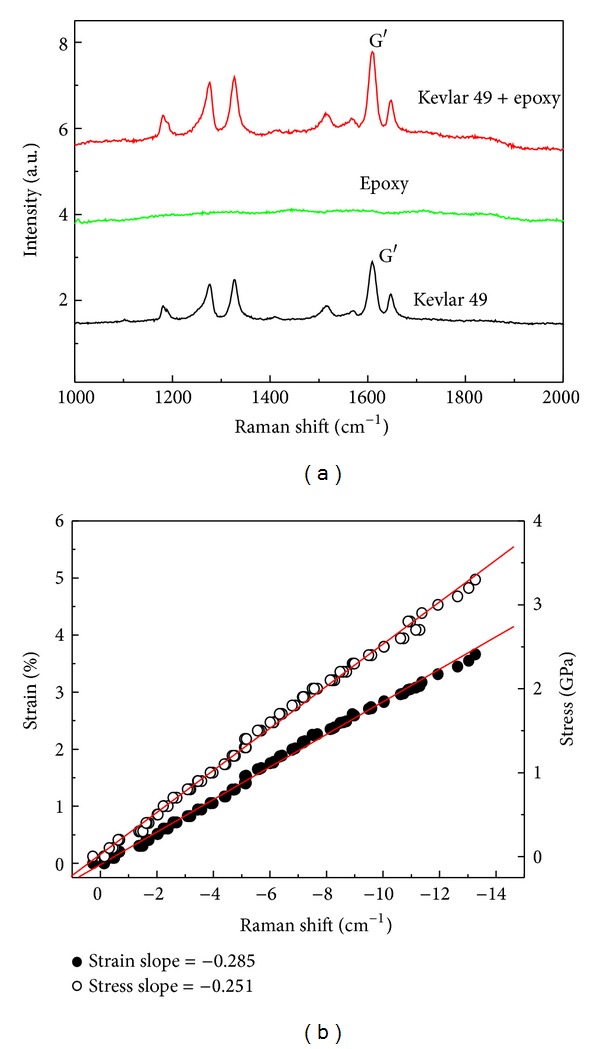
Raman spectra of (a) fiber/epoxy droplet specimen compared with pure epoxy and Kevlar 49 fiber [14] and (b) relationships of Raman shift with stress and strain for Kevlar 49 fiber [15].

When the fiber is under deformation, it causes the movement and deformation of Raman spectrum [14, 15], as shown in Figure 4(a). Although the epoxy resin has a strong fluorescence effect, the Raman spectrum of fiber/epoxy specimen after fully curing shows a Raman spectrum overlay of fiber and epoxy resin, but this does not affect the identification of the fiber Raman peak. Raman shift has a linear relationship with the strain or stress of aramid fibers [15], as shown in Figure 4(b). Therefore, it is a potential method of microscale experimental mechanics, and it has recently been used to study the interfacial micromechanical behaviors in fibrous composites, such as fiber stress distribution, stress concentration, and interface integrity.

## 4. Interface Mechanics Modeling

The interfacial stress transfer behavior between the fiber and matrix in fibrous composites is a major mechanical problem including several successive stages: the interface intact bonding, interface debonding, interface completely debonding, and fiber pullout. The elastic stress transfer in bonding area and the frictional shear stress transfer in debonded area have been widely recognized. In the process of interfacial debonding and extension, the interface mechanical parameters of bonding shear stress, debonding friction shear stress, and interface debonding length continuously evolve, and the macropulling force or stress is also changed accordingly. At present, the main interface mechanics problems in fibrous composites discussed are as follows: the elastic stress transfer, partial debonding stress transfer, interface failure criterion and fiber bridging, and so on.

### 4.1. Elastic Stress Transfer and Failure

**Figure 5:**
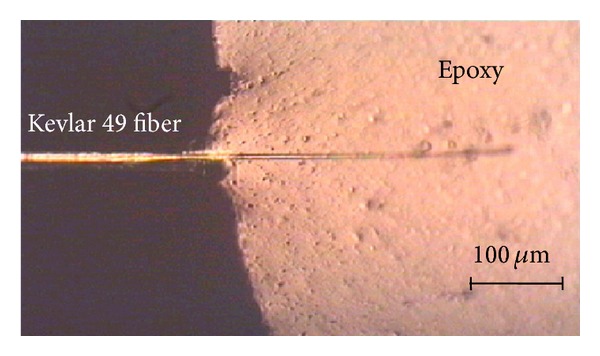
Single fiber pullout specimen [15].

One end of single fiber embeds in epoxy matrix, as shown in Figure 5, an axis tension load pulls the fiber out from the matrix. Under the assumptions of the stress uniform distribution and homogeneous isotropy, the interfacial shear strength *τ*_*b*_ can be calculated by the ratio of maximum pullout load *F*_max⁡_ and interface bonding area; namely,

(1) $$\begin{array}{c} \tau_{b}=\frac{F_{\max}}{2\pi rl}, \end{array}$$

where *r* and *l* are the fiber radius and the embedded fiber length, respectively.

The stress distribution along the embedded fiber cannot be obtained by the above equation, so it cannot be used to study the stress transfer between the fiber and matrix. Cox's shear-lag model [31] considers the force balance between the fiber axial stress *σ* and the interfacial shear stress *τ* along the embedded fiber. It satisfies the relationship as

(2) $$\begin{array}{c} \tau =-\frac{r}{2}\left(\frac{d\sigma }{dx}\right). \end{array}$$

Piggott's model [32] is further used to describe the fiber axial stress along the embedded fiber within the elastic stress transfer, so the fiber elastic stress distribution before the interface debonding is written as

(3) $$\begin{array}{c} \sigma =\sigma_{\text{app}}\frac{\sinh\left[n\left(L-x\right)/r\right]}{\sinh\left(ns\right)}, \end{array}$$

where *x* is the distance to fiber entry, *σ*_app_ is the stress acting on the fiber out of the matrix, *L* is the fiber length that the fiber axial stress decays to zero (i.e., the effective length of stress transfer), *s* is the fiber aspect ratio (*L*/*r*), and *n* is a constant related with the geometry, material parameters of fiber, and matrix [33].

**Figure 6:**
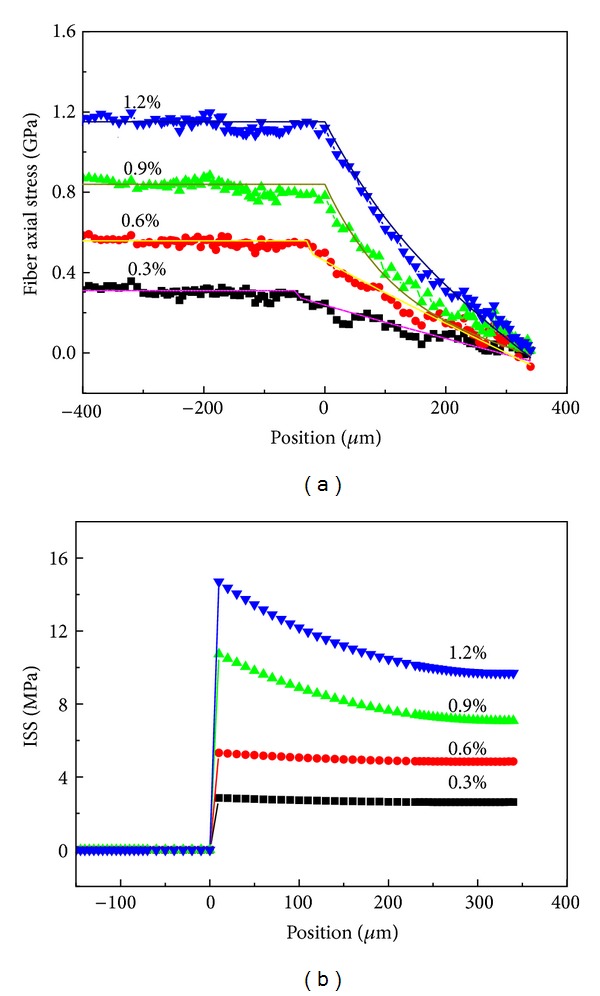
(a) Fiber axial stress and (b) shear stress distributions along fiber of pullout specimen under different strain levels [15].

Figure 6(a) shows the fiber axial stress distribution under different strain levels in fiber pullout test. It can be seen that the fiber axial stress increased significantly with the applied strain and the constant fiber axial stress out of the matrix (*x* ≤ 0) equal to the applied load; that is, *σ* = *σ*_app_. Then, the fiber axial stress along the embedded fiber was gradually reduced from the fiber entry (*x* = 0) to the embedded fiber end (*x* = *L*) in accordance with the theoretical results (solid line) of (3). In the current 1.2% strain level, the debonding phenomenon did not occur at the embedded fiber and the entire embedded fiber was under a certain load, so the intact elastic stress transfer was presented on the fiber/matrix bonding interface.

The interfacial shear stress (ISS) along the embedded fiber is further given from (2) and (3) as

(4) $$\begin{array}{c} \tau =\sigma_{\text{app}}\frac{n\cosh\left[n\left(L-x\right)/r\right]}{2\sinh\left(ns\right)}. \end{array}$$

As shown in Figure 6(b), the ISS distribution increased with the strain levels. The ISS of fiber out of the matrix was zero and reached the maximum at the fiber entry.

In the fiber pullout experiment, the aspect ratio *n* of the embedded fiber is large. The fiber stress and shear stress at the fiber entry (*x* = 0) are given by the combination of (3) and (4) as

(5) $$\begin{array}{c} \sigma_{m}=\sigma ,\;\;\tau_{m}=\frac{n}{2}\sigma . \end{array}$$

If the applied strain continues, the fiber fracture failure occurs when the fiber stress *σ* on the free fiber segment is over the fiber stress strength *σ*_*b*_. Similarly, the interfacial debonding failure occurs when the maximum ISS of *τ*_*m*_ at the fiber entry exceeds the shear strength *τ*_*b*_. Then, the strength failure conditions depending on the balance of fiber strength *σ*_*b*_ and interfacial shear strength *τ*_*b*_ are written as

(6) $$\begin{array}{c} \sigma =\frac{2\tau_{m}}{n}\geq \sigma_{b},\;\text{Fiber fracture},\\ \tau_{m}=\frac{n\sigma }{2}\geq \tau_{b},\;\text{Interface debond.} \end{array}$$

It can be seen that the fiber/matrix interface is more likely to fail if the fiber strength *σ*_*b*_ increases, and the fiber tends to break if the interface shear strength *τ*_*b*_ increases.

### 4.2. Frictional Shear Stress Transfer

**Figure 7:**
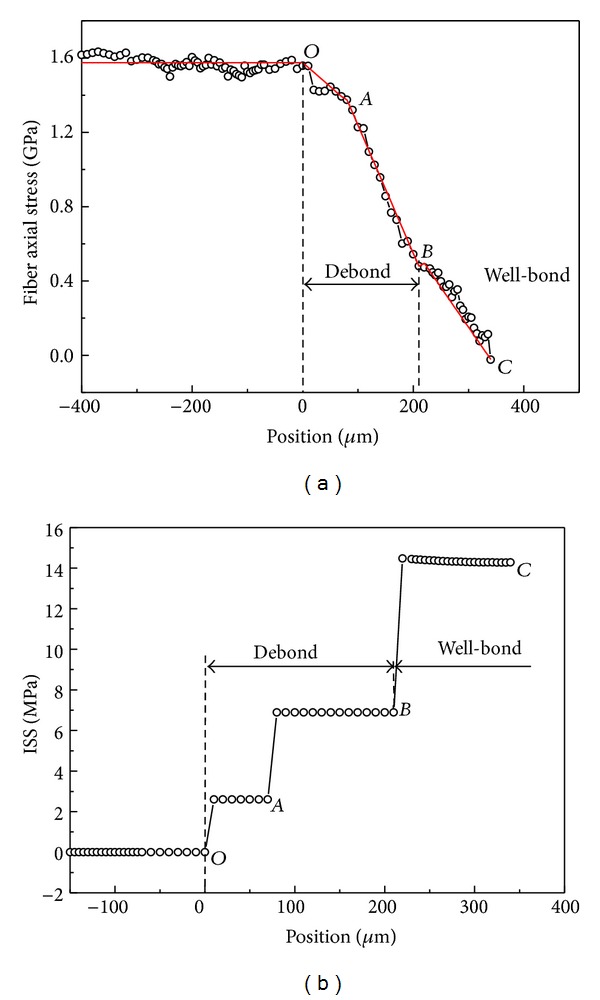
(a) Fiber axial stress and (b) shear stress distributions along fiber of pullout specimen under 1.6% strain [15].

When the applied load further increased in the fiber pullout test (Figure 5), the interface debonding failure occurred firstly at the fiber entry and then propagated along the fiber/matrix interface. The fiber axial stress distribution in Figure 7(a) shows that the fiber has debonded from the fiber entry (Point *O*) to the debonding/bonding transition (Point *B*), and the interface frictional shear stress existed on the different stages (Figure 7(b)). This is because the debonding segments of *OA* and *AB* exhibit different interface microstructures resulting in unequal shear friction effect. The interface frictional shear stress accords with the linear distribution assumption on the debonding segments (the solid lines in Figure 7(a)). After the debonding/bonding transition (Point *B*), the fiber bonding interface is still intact and the fiber axial stress distribution satisfies with the Piggott's model (Segment *BC*).

Using the simple Cox's shear-lag model, the frictional stress transfer in the debonding interface can be easily analyzed. Assuming a linear distribution of the interfacial friction stress, a two-stage model of the interfacial friction shown in Figure 7(b) gives the fiber stress distributions on the debonding interface as

(7) σOA=σapp−2τ−OAxr, 0≤x≤LOA,σAB=σapp−2τ−OALOAr −2τ−AB(x−LOA)r, LOA<x≤LOB,

where *L*_*OA*_ is the debonding fiber length on the first stage, *L*_*OB*_ is the total length of the debonding fiber, ${\overset{-}{\tau }}_{OA}$ and ${\overset{-}{\tau }}_{AB}$, respectively, correspond to the first and second stages of the interfacial friction shear stress constant, and *x* is the distance to the fiber entry (Point *O*). Piggott's model can be used to describe the fiber axial stress at the intact bonding interface (Segment *BC* in Figure 7). The fiber axial stress equals the fiber stress at the debonding/bonding transition (Point *B*), which can be obtained by solving (7) under the condition of *x* = *L*_*OB*_.

The interface frictional shear stresses on the debonding segments are given by the combination of (2) and (7) as

(8) $$\begin{array}{c} \tau_{OA}={\overset{-}{\tau }}_{OA},\;0\leq x\leq L_{OA},\\ \tau_{AB}={\overset{-}{\tau }}_{AB},\;L_{OA}<x\leq L_{OB}. \end{array}$$

It can be seen that the frictional shear stress plays the role of stress transfer on the debonding interface and can be described as the multistage constant distribution in this study.

If the load continues to be applied, the interface debonding failure propagates forward. According to strength failure conditions (6), the fiber breakage failure occurred until the maximum fiber stress (*σ* = *σ*_app_) reached the fiber strength *σ*_*b*_; otherwise the interfacial debonding failure will continue until the fiber is completely pulled out.

### 4.3. Reloading of Bridging Fiber

**Figure 8:**
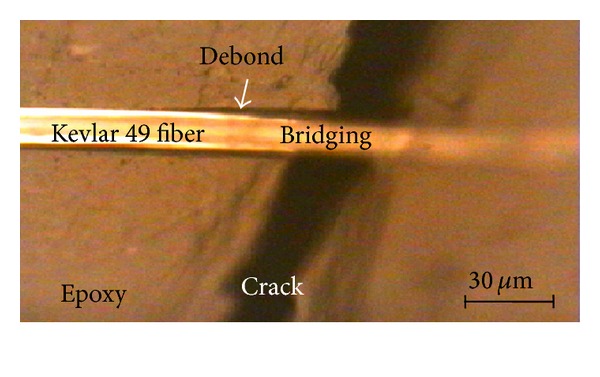
Bridging fiber and interfacial debonding during crack opening [16].

As shown in Figure 8, when a matrix crack vertically propagated across an embedded fiber without fiber breakage, the bridging fiber with partial debonding was across both sides of the matrix crack. The formation of bridging fiber can be regarded as two fibers pullout process. The bridging fiber contains three parts: the bonding segment, debonding segment, and bridging segment. The fiber axial stress meets Piggott's model in the bonding segment. It is affected by linear friction shear stress in the debonding segment and remains a constant in the bridging segment. In the following text, the interfacial stress transfer and failure conditions of the bridging fiber are considered to be reloading.

#### 4.3.1. Slip Transform and Reloading

**Figure 9:**
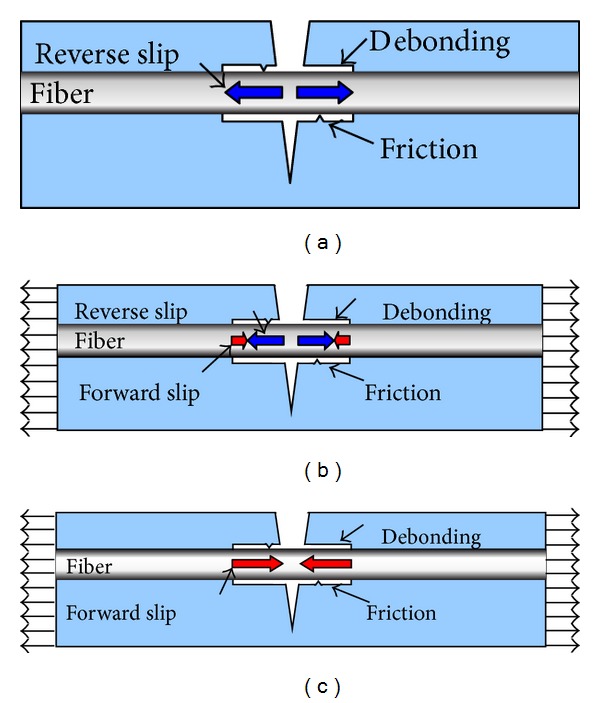
Slip transform for bridging fiber after reloading [16].

For the case of unloading after the formation of bridging fiber, a reverse slip will occur on the debonding segment and the fiber retraction results in residual interfacial friction stress, as shown in Figure 9(a). When the bridging fiber is reloaded, the partial slip on the debonding segment inverses its sliding direction. This will cause the different effects of interface friction force on the debonding segment, as shown in Figures 9(b) and 9(c).

The reverse slip happens on the fiber debonding segment before reloading (Figure 9(a)), generating the interfacial friction in the opposite direction and resulting in compressive residual stress in the debonding segment. When the load is applied again (Figure 9(b)), the partial reverse slip on the debonding segment transforms to the forward slip resulting in the reduction of reverse slip length until all reverse slip completely converses to the forward slip (Figure 9(c)). The interfacial friction in the forward slip region makes the increase of fiber stress; on the contrary, the reverse slip results in the decrease of fiber stress. It is noted that the fiber stress remains constant in the bridging segment.

#### 4.3.2. Stress Transfer Model

**Figure 10:**
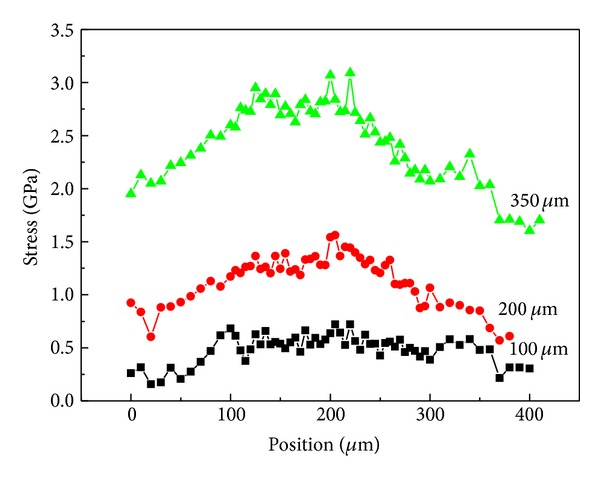
Stress distributions on the bridging fiber under different loads [16].

Raman measurements along the bridging fiber in Figure 8 gave a symmetrical axial stress distribution, as shown in Figure 10. The fiber axial stress is increased with the applied load. The stress platform is close to the bridging segment and the interfacial friction force in the slip segment should be overcome.

**Figure 11:**
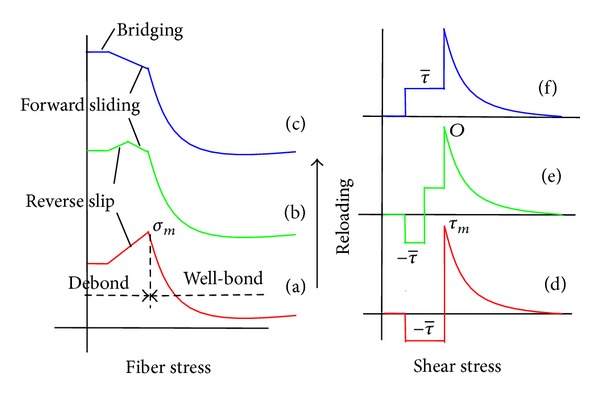
Stress transfer model of bridging fiber under reloading.

During the reloading of bridging fiber, the reverse slip in debonding segment gradually transformed into the forward slip so that the debonding fiber reloaded until the fiber stress eventually reached the maximum in the bridging segment. In fiber bridging segment, the ISS is zero due to the constant fiber stress. Setting a positive constant $\overset{-}{\tau }$ of the interfacial friction shear stress and the fiber stress *σ*_*m*_ at the bonding/debonding transition point, a stress transfer model for the bridging fiber is shown in Figure 11.

In the fiber debonding segment, the interfacial frictional shear stress (Figures 11(d)–11(f)) is a constant; namely,

(9) $$\begin{array}{c} \tau =\pm \overset{-}{\tau }, \end{array}$$

where the interfacial frictional shear stress takes a positive sign in the forward slip zone and a negative sign in the reverse slip zone. The fiber stress in the debonding segment meets a linear distribution of the interfacial friction shear stress as

(10) $$\begin{array}{c} \sigma =\sigma_{m}+2\overset{-}{\tau }\frac{x_{1}}{r}-2\overset{-}{\tau }\frac{x_{2}}{r}, \end{array}$$

where *r* is the fiber radius, *x*_1_ and *x*_2_ are the forward slip length and the reverse slip length in the debonding segment, respectively.

At initial reloading stage (Figure 11(a)), the fiber stress in the debonding segment is reduced to overcome the reverse interface friction (Figure 11(d)) due to the whole debonding segment belonging to the reverse slip zone. By contrast, the fiber stressing the bridging segment keeps constant due to no interface frictional on the bridging segment.

At middle reloading stage, (Figure 11(b)), the partial reverse slip transforms into the forward slip (Figure 11(e)). The fiber stress in the forward slip zone increases to overcome the positive interface friction so that the partial debonding fiber is reloaded.

At completely reloading stage (Figure 11(c)), the debonding fiber is completely reloaded due to the whole debonding segment belonging to the forward slip zone (Figure 11(f)).

It can be predicted that the debonding interface will continue to extend if the ISS at the bonding/debonding transition point reaches the interfacial shear strength. With the further increase of reloading, the fiber stress in the maximum stress plateau region will reach the fiber tensile strength so that the bridging fiber will fracture. This is the strength criteria for the bridging fiber.

For fibrous composites with stable interface, it can be seen from the above analysis that the physical and chemical nature of the interface determines the interface bonding ability, namely, the interface shear strength. The matrix crack across the fiber will cause interfacial debonding and form the bonding segment, debonding segment, and bridging segment. The fiber stress transfer among these segments has relationship with the interface bonding performance, interface friction, interfacial shear strength, and fiber strength. The balance between them determines whether the bridging fiber is stable or unstable. Once the balance is broken, the bridging fiber cannot exist stably and then transforms into the broken fiber [34]. The strength criteria for the bridging fiber can be used to explain the phenomenon that some of the debonding fibers can form a stable bridge but some of them break.

## 5. Remarks

The interfacial mechanical design problems faced in fibrous composites elaborated from three ways of the material optimization, interface optimization, and computational optimization. The physical, chemical, geometric, and mechanical properties at microscale have a great impact on the interface behaviors. They are necessary to develop new experimental methods for reasonable evaluation on fiber/matrix interface by fine experimental testing and characterization to improve the interface micromechanical model. Micro-Raman spectroscopy was used to study main mechanical problems in fibrous composites, including the elastic stress transfer and failure criteria of well-bonding fiber, the frictional shear stress transfer behavior of partially debonded fiber, the slip transformation, and stress transfer models of bridging fiber during reloading. These works show that micro-Raman spectroscopy has ability to evaluate the stress transfer behavior of fiber/matrix interface.

## References

[1] Baker A, Dutton S, Kelly DW (2004). *Composite Materials for Aircraft Structures*.

[2] Gibson RF (2011). *Principles of Composite Material Mechanics*.

[3] Jones RM (1998). *Mechanics of Composite Materials*.

[4] Kang YL (1999). Experimental analysis for some interfacial mechanics problems. *Mechanical Engineering*.

[5] Kim BW, Nairn JA (2002). Observations of fiber fracture and interfacial debonding phenomena using the fragmentation test in single fiber composites. *Journal of Composite Materials*.

[6] Yang XG, Wu QL (2008). *Analysis and Application of Raman Spectroscopy*.

[7] Kang YL, Qiu Y, Lei ZK, Hu M (2005). An application of Raman spectroscopy on the measurement of residual stress in porous silicon. *Optics and Lasers in Engineering*.

[8] Lei ZK, Kang YL, Cen H, Hu M, Qiu Y (2005). Residual stress on surface and cross-section of porous silicon studied by micro-raman spectroscopy. *Chinese Physics Letters*.

[9] Lei ZK, Kang YL, Cen H, Hu M (2006). Variability on raman shift to stress coefficient of porous silicon. *Chinese Physics Letters*.

[10] Qiu W, Li Q, Lei ZK, Qin QH, Deng WL, Kang YL (2013). The use of a carbon nanotube sensor for measuring strain by micro-Raman spectroscopy. *Carbon*.

[11] Qiu W, Kang YL, Lei ZK, Qin QH, Li Q, Wang Q (2010). Experimental study of the Raman strain rosette based on the carbon nanotube strain sensor. *Journal of Raman Spectroscopy*.

[12] Nishikawa M, Okabe T, Takeda N (2008). Determination of interface properties from experiments on the fragmentation process in single-fiber composites. *Materials Science and Engineering A*.

[13] Wang XH, Zhang BM, Du SY, Wu YF, Sun XY (2010). Numerical simulation of the fiber fragmentation process in single-fiber composites. *Materials and Design*.

[14] Lei ZK, Qiu W, Kang YL, Liu G, Yun H (2008). Stress transfer of single fiber/microdroplet tensile test studied by micro-Raman spectroscopy. *Composites A*.

[15] Lei ZK, Wang Q, Qiu W (2013). Stress transfer of Kevlar 49 fiber pullout test studied by micro Raman spectroscopy. *Applied Spectroscopy*.

[16] Lei ZK, Wang Q, Qiu W (2013). Micromechanics of fiber-crack interaction studied by micro-Raman spectroscopy: bridging fiber. *Optics and Lasers in Engineering*.

[17] Zhandarov S, Mäder E (2005). Characterization of fiber/matrix interface strength: applicability of different tests, approaches and parameters. *Composites Science and Technology*.

[18] Carlsson LA, Adams DF, Pipes RB (2002). *Experimental Characterization of Advanced Composite Materials*.

[19] Galiotis C, Paipetis A, Mansion C (1999). Unification of fibre/matrix interfacial measurements with Raman microscopy. *Journal of Raman Spectroscopy*.

[20] Anagnostopoulos G, Parthenios J, Galiotis C (2008). Thermal stress development in fibrous composites. *Materials Letters*.

[21] Day RJ, Rodrigez JVC (1998). Investigation of the micromechanics of the microbond test. *Composites Science and Technology*.

[22] Lei ZK, Wang Q, Kang YL, Qiu W, Pan XM (2010). Stress transfer in microdroplet tensile test: PVC coated and uncoated Kevlar-29 single fiber. *Optics and Lasers in Engineering*.

[23] Tandon GP, Pagano NJ (1998). Micromechanical analysis of the fiber push-out and re-push test. *Composites Science and Technology*.

[24] Sinclair R, Young RJ, Martin RDS (2004). Determination of the axial and radial fibre stress distributions for the Broutman test. *Composites Science and Technology*.

[25] Pitkethly MJ, Favre JP, Gaur U (1993). A round-robin programme on interfacial test methods. *Composites Science and Technology*.

[26] Zheng BL, Ji X (2002). Stress singularity analyses of interface ends in micro-mechanics tests. *Composites Science and Technology*.

[27] Ji X, Dai Y, Zheng BL, Ye L, Mai YW (2003). Interface end theory and re-evaluation in interfacial strength test methods. *Composite Interfaces*.

[28] Cen H, Kang YL, Lei ZK, Qin QH, Qiu W (2006). Micromechanics analysis of Kevlar-29 aramid fiber and epoxy resin microdroplet composite by Micro-Raman spectroscopy. *Composite Structures*.

[29] Xu LR, Kuai HC, Sengupta S (2005). Free-edge stress singularities and edge modifications for fiber pushout experiments. *Journal of Composite Materials*.

[30] Zhandarov S, Mäder E (2005). Characterization of fiber/matrix interface strength: applicability of different tests, approaches and parameters. *Composites Science and Technology*.

[31] Cox HL (1952). The elasticity and strength of paper and other fibrous materials. *British Journal of Applied Physics*.

[32] Piggott MR (1980). *Load Bearing Composites*.

[33] Nairn JA (1997). On the use of shear-lag methods for analysis of stress transfer in unidirectional composites. *Mechanics of Materials*.

[34] Lei ZK, Wang Q, Qiu W (2013). Micromechanics of fiber-crack interaction studied by micro-Raman spectroscopy: broken fiber. *Optics and Lasers in Engineering*.

